# Heat stress monitoring based on heart rate measurements

**DOI:** 10.47626/1679-4435-2020-449

**Published:** 2020-12-11

**Authors:** Alvaro Cesar Ruas, Paulo Alves Maia, Rodrigo Cauduro Roscani, Daniel Pires Bitencourt, Fabiano Trigueiro Amorim

**Affiliations:** 1 Pesquisa, Fundação Jorge Duprat Figueiredo de Segurança e Medicina do Trabalho - Campinas (SP), Brazil; 2 Health, Exercise and Sports, University of New Mexico - Albuquerque (NM), USA

**Keywords:** heat exposure, heart rate, heat stress

## Abstract

Currently, occupational heat exposure is usually measured using environmental variables such as the wet bulb globe temperature index. The costs of heat stress monitoring include the acquisition of specialized equipment and the recruitment of trained personnel. In rapidly changing environments, such as outdoor settings, these assessments must be conducted on a daily basis. The wet bulb globe temperature index has been criticized as a measure of heat stress for its failure to account for individual differences in susceptibility to heat stress, age, body mass index, physical fitness, clothing, illnesses and use of alcohol or drugs. The objective of this study was to assess the relationship between heart rate and body temperature in heat-exposed workers to determine whether heart rate can be used to monitor and prevent heat stress and physiological strain. This study was based on previous literature as well as physiological and environmental data collected from 10 individuals engaged in heavy physical labor. Heart rate, which has been recommended by the American Conference of Governmental Industrial Hygienists (ACGIH) as a possible measure of heat stress, follows a similar trend to body temperature with a slight temporal delay. Heart rate monitors with alarm systems could be developed to notify workers when to slow down their activities or take a break for thermal recovery, thereby contributing to the prevention of heat-related illness.

## INTRODUCTION

Exposure to extreme heat is a public and occupational health concern. The incidence of illness and death during summer months increases every year. Even in other seasons, death rates rise sharply in heat waves, which are currently more frequent, longer-lasting and widespread than ever before.^1^ This issue is aggravated by global warming, which creates environmental conditions that are increasingly threatening to human and animal health.^2^

Heat exposure in occupational environments affects worker health and productivity, and increases the likelihood of occupational accidents.^3^^-^^5^ This, in turn, has a negative impact on household consumption rates, social inequality and the gross domestic product (GDP).^6^ Excess heat exposure can be prevented in several ways, the most important of which is monitoring. Currently, the most common method of heat exposure monitoring involves the assessment of environmental parameters, such as natural wet bulb temperature, globe temperature and dry bulb temperature, which can be used to calculate the wet-bulb globe temperature (WBGT) index. The WBGT index is the most well-known international measure of occupational heat exposure, and is used to evaluate the exposure of groups of workers, regardless of individual susceptibility to heat stress.^7^

Additional variables considered in the assessment of heat exposure include individual characteristics such as age, gender, body mass index (BMI), physical conditioning, clothing, illnesses, alcohol intake, smoking status, etc. Current regulations on the subject^7^^,^^8^ classify activities into intervals according to metabolic demands, thereby calculating the amount of heat generated by physical activities of a similar type. The assessment of heat exposure may also represent a significant cost to employers, as it requires the acquisition of specialized equipment and the recruitment of trained personnel such as engineers, physicians and technicians, as required by legislation on occupational health and safety.^8^

In external environments, with large variations in temperature, humidity, radiation and wind speed, heat exposure should be constantly monitored throughout the workday, especially in the case of physically demanding activities. However, this is a costly procedure and therefore rarely performed, even in the hottest months of the year. This is one of the main reasons for the high rates of heat-related illnesses and heatstroke death in rural setting.^9^^,^^10^ A more precise assessment of heat exposure in the workplace can be obtained using methods of occupational monitoring based on physiological parameters such as core body temperature (CBT), skin temperature, heart rate (HR), sweat rate or algorithms based on two or three of these variables.^11^^-^^15^ Some studies have also combined the assessment of environmental and physiological variables.^5^^,^^16^^-^^20^

According to the American Conference of Governmental Industrial Hygienists (ACGIH), heat stress is defined by the presence of any of the following conditions^15^: HR, in beats per minute (bpm), remains for several minutes above the value obtained by subtracting the individual’s age (in years) from 180 bpm; CBT is over 38.5°C for acclimatized and medically selected individuals or over 38°C for unacclimatized, unselected individuals; recovery HR at 1 minute after peak physical effort exceeds 120 bpm; there are symptoms of severe and sudden fatigue, nausea, vertigo or dizziness. Moran,^12^ who developed the physiological strain index (PSI) based on both rectal temperature and HR, found that the former follows a similar trend as the latter. In experiments involving 100 young adults, a rectal temperature of 38.0°C was associated with a HR of 140 bpm, while a temperature of 38.6°C was related to a HR of 59 bpm.

The aim of this study was to assess the relationship between HR and body temperature in sugarcane cutters under normal outdoor working conditions, where all participants were physically able to perform their duties as determined by clinical examination. This was done to collect evidence to support the use of HR as a measure of heat and physiological stress. If HR is confirmed to provide a suitable estimate of heat stress, it could be monitored and used to alert workers of the need to take measures to cool off when needed, avoiding exposure to health risks.

## METHOD

The research question was investigated using findings from previous studies and assessments of physiological and environmental data collected from individuals engaged in heavy physical labor. The study involved 10 volunteers aged 20 to 40 years recruited from an agricultural company in the state of São Paulo. The volunteers had at least 1 year of experience in manual sugarcane cutting, working from 8:00 to 15:00 every day, 7 hours a day, for a total of 42 hours a week. During harvest season, all workers remained in the fields, where they slept and took all meals. All participants provided written consent after having received information about the goals of the study, the procedures involved and the ethical precautions taken in the study. This investigation was approved by a research ethics committee (Certificate of Appreciation for Ethical Consideration No. 27690014.0.0000.5108). Data were collected from October 17th to 29th, 2015, in two sugarcane plantations in the state of São Paulo.

All volunteers had previously completed a clinical assessment at the Santa Casa de Misericórdia hospital in the city of São Paulo. Subsequently, after a rest period of 2 days, all participants underwent an anthropometric assessment and progressive treadmill test. Weight and height were measured to the nearest 0.1 kg and 0.5 cm using a Welmy 110 (Brazil) balance beam scale with attached stadiometer. HR was measured using a Polar RS800 monitor (Polar Electro, Finland). Preliminary testing was conducted to calculate the maximum HR of each volunteer. All tests were terminated due to fatigue, with no patients reporting dizziness, nausea, blurred vision, dyspnea or precordial pain. Test results were considered maximum when at least three of the following criteria were met: respiratory exchange ratio > 1.15; perceived exertion score over 18 on the Borg,^21^ scale; HR > 90% of the maximum age-predicted HR (HRMax = 220 - age) and volitional fatigue.^22^

CBT was measured using a Vitasense Model VSM100M (Mini-mitter Company, Inc., Bend, OR) radiotelemetry capsule in the gastrointestinal tract, since this method provides the most accurate measure of internal temperature.^23^ Heat exposure was evaluated using the Heat Stress software package developed by Fundacentro.^24^ The software uses meteorological data collected automatically from the National Meteorology Institute (Instituto Nacional de Meteorologia; INMET) to calculate the hourly WBGT index for any region in the country. This index is used by both the International Organization Standardization (ISO) and Brazilian authorities^8^ to assess extreme heat exposure. For outdoor settings with sun exposure, the WBGT index is calculated using the following equation^25^:

WBGTindex=0.7nbt+0.2gt+0.1sbt

Where gt is the globe temperature, nbt is the natural wet bulb temperature and sbt, the air temperature.

Field data were collected using HR monitors worn by participants throughout their shifts on 2 consecutive days. At lunch time, the devices were powered off. On day 1, participants worked at a normal pace, while on day 2, they were asked to work at approximately 75% of their normal speed. This reduction was monitored based on the number of meters of sugarcane harvested - to this end, a researcher would ask participants to work faster or slower, as needed, in order to reach the predetermined target by the end of the day.

The age and anthropometric measurements of study participants are shown in Table 1. The data in the table shows that participants varied widely in age, with a mean of 26.6 years (SD, 6.7 years). The age difference between the youngest and oldest participants was 21 years. All participants were in the young adult age group, with mean height and weight very similar to the estimated mean values for a standard adult male (70 kg and 1.70 m), as specified in the ISO 8996,^26^ standard which describes the metabolic heat generated by different types of activity.

**Table 1:** Age and anthropometric measurements of participants

	Age (years)	Weight (kg)	Height (m)
Worker identification			
1	40	71.0	1.70
2	24	68.0	1.62
3	34	57.8	1.72
4	31	72.5	1.73
5	19	66.4	1.52
6	20	55.3	1.60
7	22	85.9	1.79
8	24	85.5	1.65
9	24	72.8	1.69
10	28	76.0	1.69
Mean	26.6	71.1	1.67
Standard deviation	6.7	10.1	0.08

Tables 2 and 3 show the maximum CBT (BT1 and BT2), HR (H1 and H2) and heat exposure for each worker as measured by the WBGT index on each day of data collection. The association between these values and the age of participants, their productivity (Prod1, Prod2 and Prod2/Prod1), in meters of sugarcane cut, and maximum allowable HR (HRmax) was then examined. HRmax was calculated using the following equation, as recommended by the ACGIH^15^:

HRmax=180−age

**Table 2:** Physiological and environmental data and daily productivity of sugarcane cutters (Prod2/Prod1 > 75%)

Worker	Age (years)	CBT1 (°C)	CBT2 (°C)	HR1 (bpm)	HR2 (bpm)	HRmax (bpm)	WBGT1 (°C)	WBGT2 (°C)	Prod1 (m)	Prod2 (m)	Prod2/Prod1 (%)
1	40	39.0	38.7	161	170	140	27.5	28.6	136	125	92
3	34	38.1	38.0	145	125	146	28.5	27.9	90	87	97
4	31	38.8	38.1	160	128	149	28.5	27.9	77	70	91
7	22	38.8	38.3	158	138	158	29.0	28.2	121	106	88
10	28	-	38.4	149	135	152	28.3	25.3	130	142	109
Mean	31.0	38.7	38.3	155	139	149	28.4	27.6	111	106	95
SD	6.7	0.4	0.3	7.2	18.0	6.7	0.5	1.3	25.9	28.8	8.3

bpm: beats per minute; CBT1: maximum core temperature on day 1; CBT2: maximum core temperature on day 2; HR1: maximum heart rate on day 1; HR2: maximum heart rate on day 2; HRmax: maximum recommended heart rate; Mean: arithmetic mean; Prod1: productivity on day 1; Prod2: productivity on day 2; SD: standard deviation; WGBT1: maximum wet-bulb globe temperature index on day 1; WGBT2: maximum wet-bulb globe temperature index on day 2; Worker: worker identification.

**Table 3:** Physiological and environmental data and daily productivity of sugarcane cutters (Prod2/Prod1 ≤ 75%)

Worker	Age (years)	CBT1 (°C)	CBT2 (°C)	HR1 (bpm)	HR2 (bpm)	HRmax (bpm)	WBGT1 (°C)	WBGT2 (°C)	Prod1 (m)	Prod2 (m)	Prod2/Prod1 (%)
2	24	39.2	38.2	156	152	156	28.6	27.5	201	100	50
5	19	38.1	38.0	-	125	161	28.4	27.5	76	57	75
6	20	38.5	38.2	172	150	160	27.5	28.5	95	56	59
8	24	38.6	38.6	161	160	156	27.0	29.0	166	94	57
9	24	38.3	-	140	148	156	25.2	28.2	177	112	63
Mean	22.2	38.5	38.3	157	147	158	27.3	28.1	143	84	61
SD	2.5	0.4	0.3	13.3	13.1	2.5	1.4	0.7	54.4	25.8	9.2

bpm: beats per minute; CBT1: maximum core temperature on day 1; CBT2: maximum core temperature on day 2; HR1: maximum heart rate on day 1; HR2: maximum heart rate on day 2; HRmax: maximum recommended heart rate; Mean: arithmetic mean; Prod1: productivity on day 1; Prod2: productivity on day 2; SD: standard deviation; WGBT1: maximum wet-bulb globe temperature index on day 1; WGBT2: maximum wet-bulb globe temperature index on day 2; Worker: worker identification.

The analysis of productivity ratios between days 2 and 1 (Prod2/Prod1) showed that five of the 10 workers (Workers 1, 3, 4, 7 and 10) did not reduce their pace by 25% on study day 2, as requested by the researchers, while others slowed down even more than requested (Workers 2, 6, 8 and 9). Their data were therefore analyzed in two separate groups, shown in Tables 2 and 3, respectively. The tables show that the WBGT ranged from 25.3 to 29.0 °C, which indicates potentially hazardous heat loads on both study days, requiring the implementation of temperature control measures such as pauses for thermal stabilization. Under theses environmental conditions, six workers who attained their HRmax (Workers 1, 2, 4, 6, 7 and 8) also registered temperatures of at least 38.5°C, the maximum limit recommended by the ACGIH.^15^ The fact that both HR and CBT signaled the presence of heat stress indicates an association between the two variables. his was especially evident on normal productivity days, though it was also observed during reduced productivity in Worker 8, for whom Prod2/Prod1 = 57%, CBT1 = CBT2 and HR2 = HR1-1. This may be because WBGT2 was 29 °C while WBGT1 was 27 °C, suggesting greater heat exposure on day 2, when productivity was lower. A similar pattern was observed for Worker 1, although in this case, Prod2/Prod1 = 92%, CBT2 = CBT1 -0.3 and HR2 = HR1 + 9.

Table 4 shows that, on both test days, morning productivity (P_1morning_ and P_2morning_) was usually higher than that observed in the afternoon (P_1afternoon_and P_2afternoon_). On average, the morning accounted for 64.5% of total productivity on day 1 (P_1morning_/Prod1) and 69.5% on day 2 (P_2morning_/Prod2). The decreased productivity in the afternoons may be the result of physical fatigue from the morning’s work as well as the environmental effects of higher afternoon temperatures. Though this is not shown in the table, the researchers observed that half the participants worked at a faster pace in the 1st hour of their shifts, reaching peak productivity and subsequently slowing their pace. While laborers may be encouraged to work faster if their wages depend on their productivity, it must be noted that even with maximum effort, productivity is ultimately determined by other variables, such as weather conditions, cutting techniques and the type of equipment available.

**Table 4:** Productivity in meters of sugarcane cut on the morning and afternoon of the 2 test days

Worker	Prod1	P_1morning_	P_1afternoon_	Prod2	P_2morning_	P_2afternoon_
1	136	104	32	125	73	52
2	201	126	75	100	58	42
3	90	50	42	87.3	-	-
4	77	-	-	70	40	30
5	76	46	30	57	37	20
6	95	52	43	56	41	15
7	121	90	31	106	70	36
8	166	96	70	94	71	23
9	177	96	81	112	92	20
10	130	76	54	142	112	30
Mean	126.9	81.8	50.9	94.9	66	29.8
SD	41.4	26.1	18.8	26.8	23.8	11.2

Mean: arithmetic mean; P_1morning_: morning productivity on day 1; P_1afternoon_: afternoon productivity on day 1; P_2morning_: morning productivity on day 2; P_2afternoon_: afternoon productivity on day 2; Prod1: productivity on day 1; Prod2: productivity on day 2; SD: standard deviation; Worker: worker identification.

Under these circumstances, as in the study conducted by the National Institute for Occupational Safety and Health (NIOSH),^27^ pauses based on the real-time monitoring of physiological variables could help workers avoid elevated HR and CBT, and maintain a stable productivity throughout the day. Though this was beyond the scope of the present study, frequent pauses would also benefit the osteomuscular system.

**Figure 1:**
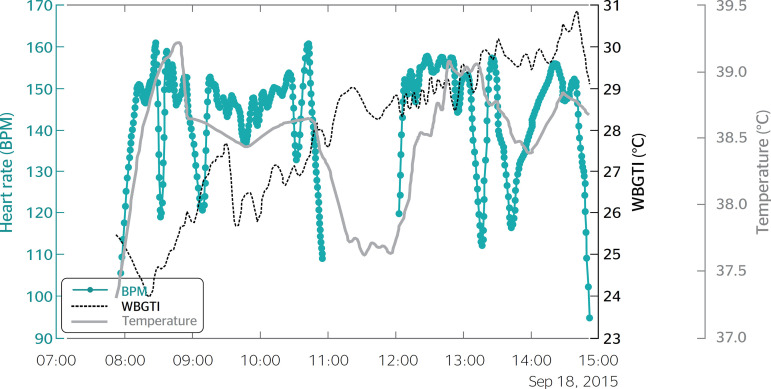
Heart rate, wet-bulb globe temperature index and body core temperature of one participant. BPM: beats per minute; WBGTI: wet-bulb globe temperature index; Temperature: body core temperature.

**Figure 2:**
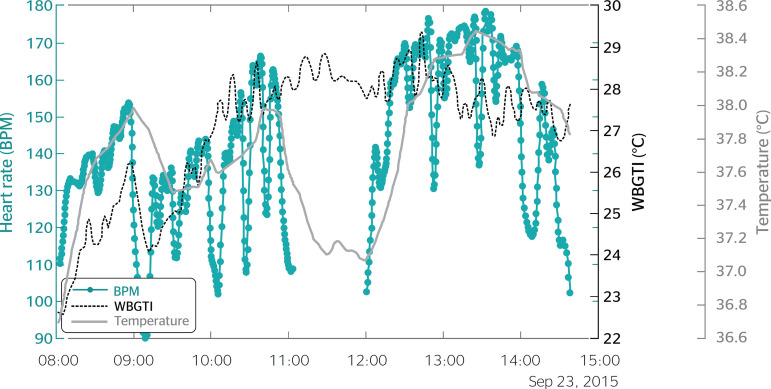
Heart rate, wet-bulb globe temperature index and body core temperature of one participant. BPM: beats per minute (heart rate); WBGTI: wet-bulb globe temperature index; Temperature: body core temperature.

Figures 1 and 2 show the HR and CBT curves observed in the data collected from two sugarcane cutters.

The curves show a series of peaks followed by steep curves from 11:00 to 12:00. Although the HR and CBT declines, suggesting that the pace at which the laborers curves look similar, the HR responds faster to changes work is not constant, and is actually determined by their in physical effort or heat exposure, which explains the physical endurance. The HR curves also present clear temporal delay between the two measurements. Similar declines followed by rapid increases, which correspond findings were also obtained by Gotshall et al.,^5^ though to brief pauses for activities such as drinking water. their experiment was conducted in a laboratory and the In this study, HR monitors were powered off during tasks involved were much less physically demanding lunch breaks, which explains the interruption in the than those involved in the present study.

The temporal delay between HR and CBT measurements in the present study ranged from 5 to 15 minutes. This is highly relevant, as it suggests that when HRmax is reached, CBT is still below the limit value of 38.5ºC stipulated by the ACGIH.^15^ Therefore, it is possible to establish HR limits for each individual, as a percentage of HRmax, which signal the need to implement measures to cool down and ensure that CBT stays within acceptable limits. A personalized monitoring device could be worn by workers and emit a sound to inform them when to slow down their pace, take breaks, step away from heat sources or drink water and rehydration salts in a shady area. In addition to preventing heat stress and related illnesses, this procedure could optimize productivity in warm environments by improving the compatibility between the activities performed and the physical capacity and heat susceptibility of individual workers.

In a study conducted by the NIOSH,^27^ HR and CBT were used to analyze heat stress in two groups of mine rescue workers. Both groups were given 1 hour to traverse a specified path; the first group completed the path in 45 minutes and rested for 15 minutes. The second group took breaks as they traveled whenever the HR of any group member reached HRmax as determined by ACGIH^15^ guidelines. Each pause lasted until the HR of the individual in question fell to within 10% of the value measured before the start of the experiment. The results showed that HR remained much lower in the group that took multiple breaks. Recovery HR was therefore determined to be a useful tool to determine work pace in order to avoid elevations in HR.

Bernard & Kenney^11^ proposed a physiological stress index based on worker HR which would allow them to assess and control their heat stress before the onset of significant symptoms. The authors conceived a personal monitor with two stages of alert. The first would signal that heat stress is increasing and the worker could not sustain their current pace for much longer; this would allow workers to slow down and continue their activities. The second stage would alert workers to stop their activities, move away from the heat exposure area and take the necessary measures to cool down. The authors established HR limits for each warning stage as a function of individual age and duration of heat exposure. This setup would require continuous monitoring, so that heat exposure could be measured based on mean HR calculated across seven intervals: 5, 10, 15, 30, 45, 60 and 90 minutes.

## CONCLUSIONS

Current evidence provides a sufficient technical basis for the use of HR as an indicator of heat stress, especially for heavy and very heavy work categories, as defined by ISO 7243.^7^ This would require the physiological monitoring and selection of workers based on their age, BMI, acclimatization, overall health status and everyday habits.

In hot environments, monitoring systems should also include a means of alerting workers when there is a risk of developing dangerously high core temperatures. An indirect but accurate method of monitoring this variable is by detecting when HR remains above the value obtained by the calculation (180 -age), since changes in HR precede changes in CBT. However, it is important to define a tolerance period during which HR is allowed to exceed this value, since some workers may experience brief peaks in HR during the work day which should not represent any danger for those in good cardiovascular health. The ACGIH,^15^ does not provide a quantitative definition for this tolerance period, stating only that one definition of heat stress involves maintaining an elevated HR for “several minutes.” The data collected in the present study did not allow for a precise estimation of this value, and no previous studies appear to provide such an estimate. There is, as such, a need for further research on this issue.

Physiological monitoring is essential for workers whose wages depend on productivity, especially in the sugarcane industry, where workers are at high risk of exceeding recommended physical activity levels. In these cases, an alarm system should indicate to workers when to reduce the pace of work and implement measures to cool down their bodies. It is important to note that workers trained to follow a monitoring program to prevent elevations in HR and CBT will need to take breaks for physical recovery during the work day, though the total duration of these pauses is shorter than that specified in relevant legislation.^8^ It is likely that the simple reduction in work pace would allow workers to carry out activities for longer periods of time without exposure to unnecessary risks.

The adoption of a physiological monitoring system such as that proposed in the present study could help prevent illness and loss of productivity, though it should be part of a more comprehensive program for the prevention of heat exposure whose actions begin with worker recruitment, by considering the age, BMI, overall health status and healthy lifestyle habits of workers. It is also important to give workers access to information and training regarding environmental conditions, especially temperature and humidity; work techniques and tool conditions; physical fitness, work wear and personal protective equipment (PPE); as well as water and hydration salt intake during work activities.

The use of HR to monitor heat stress, though possible, may face technological barriers since existing devices have not been tested in highly demanding conditions, such as frequent abrupt movements, physical impact and exposure to high levels of humidity and dust. The challenge will be to develop a device that works reliably under these conditions but is not so costly as to discourage its broader use.
